# Cu and Na contents regulate N uptake of *Leymus chinensis* growing in soda saline-alkali soil

**DOI:** 10.1371/journal.pone.0243172

**Published:** 2020-12-01

**Authors:** Hongshan Liu, Yuefen Li, Shujie Li

**Affiliations:** 1 College of Earth Sciences, Jilin University, Changchun, Jilin, China; 2 Key Laboratory of Mineral Resources Evaluation in Northeast Asia, Ministry of Land and Resources, Changchun, Jilin, China; Institute for Sustainable Plant Protection, C.N.R., ITALY

## Abstract

*Leymus chinensis (L*. *chinensis)* is the dominant plant in the eastern margins of the Eurasian temperate grasslands. It is a very robust species, exhibiting good saline-alkali resistance and stabilizing soil. In this study, 67 soil samples and *L*. *chinensis* were collected in western Jilin province, China. The contents of N, P, K, S, Mn, Fe, Zn, Cu and Na were measured, revealing that the growth of *L*. *chinensis* was mainly restricted by N based on the stoichiometric N: P ratios of plant. Furthermore, path analysis indicated that N was significantly correlated with K, S, Cu, and Zn. Imbalances in the homeostasis of these four elements may thus constrain N. The homeostasis index of Cu (*H*_Cu_) in sites with 100%-70% of vegetation cover was only 0.79, it was classified as a sensitive element. However, K, S and Zn, whose concentrations in *L*. *chinensis* were significantly related to those of N, exhibited no homeostatic characteristics. These results suggest that when seeking to treat saline-alkali stress, it is important to add fertilizers containing K, S, and Zn to avoid growth limitation. Na^+^, an ion associated with high soil alkalinity, exhibited weak homeostasis in *L*. *chinensis* even in sites with only 40%-10% of vegetation cover. When soil Na exceeded 16000 mg/kg, the homeostasis mechanism of *L*. *chinensis* appeared to be overwhelmed, resulting in rapid and probably harmful accumulation of Na. Proper control of N content can alleviate the toxicity of Na stress in *L*. *chinensis* and enhance its Na tolerance. Together, these results suggest that combined fertilization with N, K, S, Zn and Cu should be applied to improve grasslands growth. The results of this study can provide a reference basis for sustainable grassland management.

## Introduction

Soil salinization has become one of the world’s most serious environmental geological hazards. A study conducted by the FAO found that salinization is currently affecting 6 percent of the world’s land area and is degrading the limited land resources on which human beings depend [1]. Grasslands are important components of the terrestrial ecosystem; they are vital for sustainable land use and development, and for the maintenance of a dynamic balance between ecosystems [2]. The number of high quality pasture sites around the world has declined sharply in recent years because of factors such as global environmental change and overgrazing, which reduce vegetation cover and soil formation, and induce severe deterioration of grassland ecosystems. These processes have significant adverse effects on local social and economic development [3].

The western region of Jilin Province is located in northeastern China, extending over the Songnen plain in the south and the Horqin grassland in the east. It is an important region of agricultural production and animal husbandry within China but it also has one of the highest concentrations of soda saline-alkali soil in the world [4]. *L*. *chinensis*, the dominant grassland plant in this region, is highly tolerant of saline-alkali conditions [5]. Moreover, it achieves high yields and is valuable in both ecological and economic terms–the former because of its ability to stabilize grasslands, the latter because of its usefulness as fodder [6]. It has therefore been studied extensively as a pioneer plant for the improvement and utilization of saline-alkali grassland [7].

Grassland cover change is an important factor to consider when evaluating the status of grassland ecosystems and resources, and is an important indicator of grassland degradation [8, 9]. Grassland degeneration is often accompanied by reduced vegetation cover, vegetation bearing capacity, and vegetation productivity. In addition, the nutrient storage of degraded grasslands is appreciably lower than that of other ecosystems [10]. Soil elements are key raw materials for plant growth [11–14]. Changes in an element’s abundance in the soil may affect its availability to plants and alter its geochemical cycles, and may consequently limit the sustainability of agricultural production [15]. Therefore, changes in element storage are a major cause of reverse succession during grassland degradation [16]. Adverse external environmental conditions have also been shown to induce changes in plants’ elemental uptake mechanisms, leading to increased absorption of resistant elements [17]. Despite these challenges, *L*. *chinensis* thrives in such environments. Consequently, an understanding of its nutrient utilization strategy could provide insights that would help explain the relationships between different soil elements in saline-alkali grasslands with different cover levels, and may be useful in identifying ways to halt or reverse the spread of saline-alkali conditions.

Ecological stoichiometry is a theoretical concept and a field of study that has been defined as “the balance of multiple chemical substances in ecological interactions and processes, or the study of this balance” [18, 19]. Many studies on ecological stoichiometry have focused on the major nutrient elements C, N, and P [10, 20–22], but there have only been a few such studies on minor and trace nutrient elements [23, 24]. In this paper, we use the path analysis method to study the relationships between two core nutrient elements (N and P) and seven elements (K, S, Mn, Fe, Zn, Cu and Na), analyze their interactions with multiple independent variables [25], and explore the relationship between the core elements that limit the growth of *L*. *chinensis* and the levels of other elements on grasslands with different degrees of cover. The ecological stoichiometry homeostasis is one of the core theories of ecological stoichiometry. Studies have shown that plants can maintain homeostasis under abiotic stresses including soil salinization, drought and extreme temperature [26]. Nakayama has argued that plants may maintain homeostasis of mineral ions by secreting a hydrophobic barrier to regulate the horizontal diffusion of ions in the soil-plant system [27]. Similarly, Reich has argued that the regulation of elemental stoichiometry is important in the adaptation of plants to different soil environments [28]. Therefore, the homeostasis model was used to investigate elements limiting the growth of *L*. *chinensis* on grassland sites with high, intermediate, and low degrees of cover.

In the present study, we hypothesized that growth of *L*. *chinensis* was mainly restricted by either N, P, or both in saline-alkali grasslands; K, S, Mn, Fe, Cu, Zn and the interaction between elements in saline-alkali grassland soil may constrain N; the homeostasis of *L*. *chinensis* can be overwhelmed by high Na^+^. For testing these hypotheses, samples of soil and *L*. *chinensis* were collected in the west of Jilin province, China to determine which elements limit the growth of *L*. *chinensis* and to characterize the interactions between these limiting elements and other nutrient elements. We next analyzed the homeostasis of the chosen elements in *L*. *chinensis* to evaluate their influence on the growth-limiting elements. This work, and the results presented herein, broaden the scope of elemental stoichiometry, provide new insights into the saline-alkali tolerance of *L*. *chinensis*, and open a new direction for studying the degradation of saline-alkali grassland. In addition, the results obtained provide valuable guidance for the improvement and fertilization of soda saline-alkali grasslands.

## Materials and methods

### Description of the study area and selecting sampling points

The western Jilin Province is the main distribution area of soda saline-alkali soil in Songnen Plain. The study area is mainly identified as natural *L*. *chinensis* meadow [29]. The evaporation capacity greatly exceeds the precipitation. Soils of the study area have a loose texture with a higher proportion of non-capillary pores than capillary pores, and exhibit poor retention of water and fertilizer. Detailed descriptions of the study area can be found in previously published research [5].

Remote sensing data (in the form of the MOD13Q1 product) for western Jilin Province was downloaded from the website of United States Geological Survey (USGS) (http://glovis.usgs.gov). This dataset consists of MODIS NDVI images from May 2014, May 2015, and May 2016. Each image provides vegetation index values on a per-pixel basis, with a spatial resolution of 1km × 1km. To ensure consistency across the downloaded data, the MODIS Tools software was used to transform the data format. After geometric accuracy correction, radiation correction and interference factor processing, the remote sensing image data were combined with current land use maps in order to classify the degree of vegetation cover in different parts of the study area. Accordingly, the study area was subdivided into regions with vegetation coverage of 100–70%, 70–40%, 40–10% and 10–0% using the re-classification module of the ArcGIS 10.0 software package. It was discovered that the main plant species in the regions with 0-10% cover were *Puccinellia distans* and *Suaeda glauca*. Since no samples of *L*. *chinensis* could be collected from these regions, the 0–10% cover range was excluded from the study.

**Fig 1:**
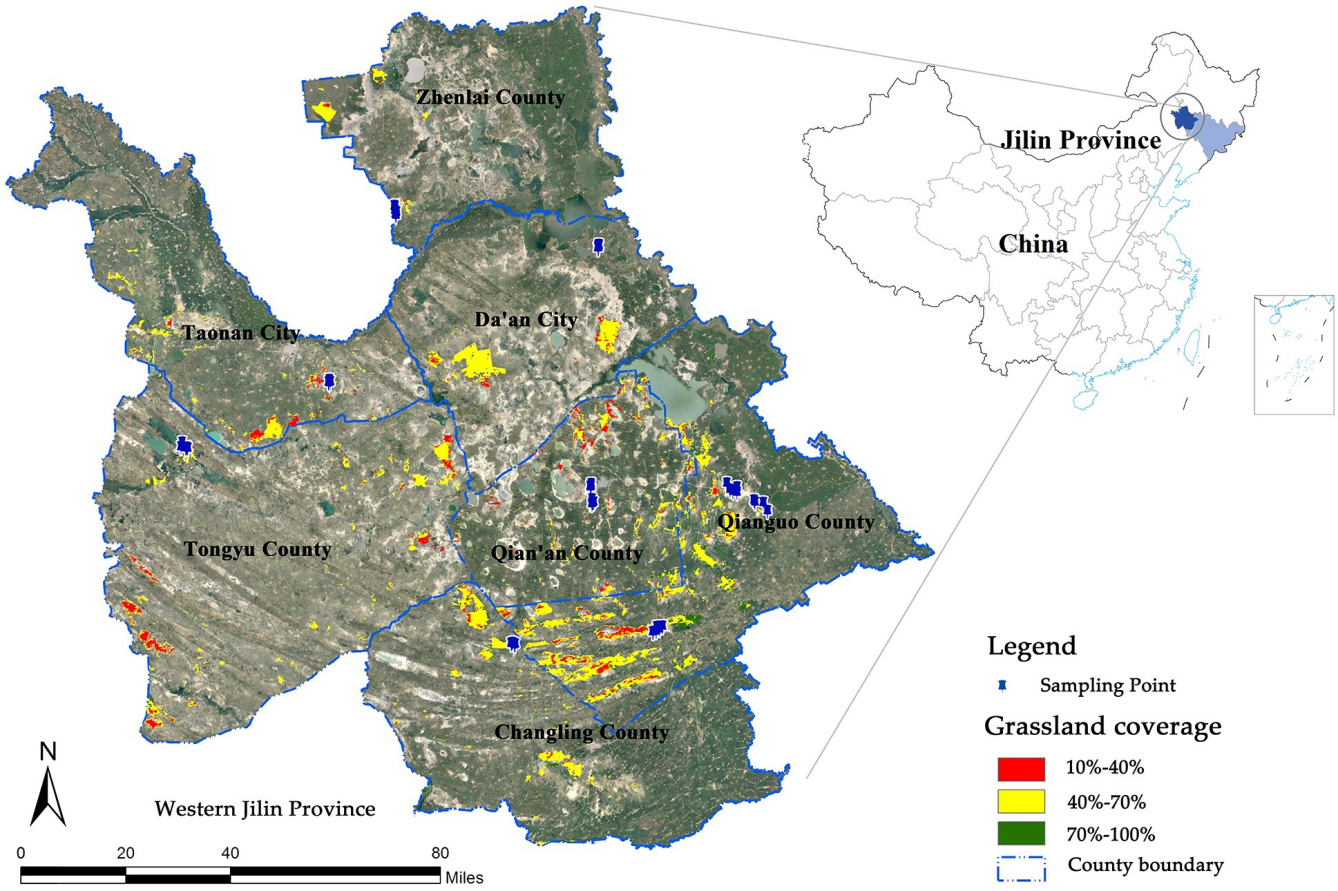
The location of the study sites (121°30′E~126°00′E, 44°00′N~46°00′N) in the western Jilin Province in Northeast China.

Sampling sites were established within western Jilin Province (Fig 1): (1) Tongyu County, 5 quadrats; (2) Taonan City, 4 quadrats; (3) Zhenlai County, 8 quadrats; (4) Da’an City, 19 quadrats; (5) Qian’an County, 9 quadrats; (6) Changling County, 6 quadrats; (7) Qianguo County, 16 quadrats. In total, 67 samples were collected. The collected samples obtained do not need a license in this study, because we selected the natural grassland, mainly the grassland growing naturally, and the grassland without any artificial improvement or artificial interference, which does not belong to any specific individual or any particular company. Therefore, there is no ownership dispute in this collection.

### Sample collection and testing methods

Considering the degree of soil salinization and density of *L*. *chinensis* [29], this investigation was based on vegetation coverage combined with soil pH and total alkalinity (CO_3_^2-^+HCO_3_^-^), as summarized in Table 1. The research team chose sampling sites that were flat, at the same elevation, and had consistent soil types [7]. Samples of soil and *L*. *chinensis* were collected on June, 2017. After locating the sampling sites using GPS, representative sampling quadrats with areas of 1m × 1m were placed at locations with suitable levels of *L*. *chinensis* cover. When collecting *L*. *chinensis* samples, the aboveground part of the plant was cut off with scissors and extraneous materials (other weeds, etc.) were removed manually. The cleaned *L*. *chinensis* samples were then placed in numbered sample bags. When collecting soil samples, we tried to avoid areas containing livestock excrement, and removed litter and dead insects from the soil surface. Soil samples were taken from the 0 to 20 cm soil layer at 5 randomly selected positions in each sampling quadrat using a 3.5 cm diameter stainless steel soil sampler. These initial samples were then carefully mixed to form a composite sample for the quadrat, which was then air-dried in the laboratory.

**Table 1:** Soil pH, total alkalinity and total Na of sampling sites.

Cover	Number of Quadrats	Soil pH	Total Alkalinity (mg/kg)	Soil Total Na (mg/kg)
100%-70%	19	7.93–9.76	409.92–1475.76	13234.67–20271.27
70%-40%	26	8.03–9.52	153.72–939.72	11975.06–20640.22
40%-10%	22	8.09–9.87	146.40–3615.66	10802.89–19234.17

The air dried soil samples were crushed and ground and sieved through 0.15mm sieve before analysis. *L*. *chinensis* samples were ground to analyze the elements. Soil total N and plant total N were assayed using a continuous flow analyzer (SAN++, Netherlands) by the LY/T 1228–2015 and LY/T 1269–1999 methods, respectively. Total P, S, Cu, Mn, Fe, K, Na, and Zn levels in the soil and *L*. *chinensis* samples were assayed using ICP-AES (ICPS-7500, Japan) by the LY/T 1256–1999 and LY/T 1270–1999 methods, respectively. Soil samples were digested in 100-mL high beakers with a mixture of 3mL concentrate nitric acid, 5mL concentrated hydrochloric acid and 2mL concentrated sulfuric acid. *L*. *chinensis* samples were digested with a mixture of nitric acid and perchloric acid (30mL). Soil total alkalinity was estimated by summing contents of CO_3_^2-^ and HCO_3_^-^. Soil pH was measured in 1:2.5 (w/v) soil distilled water extracts. Detailed descriptions of the testing methods can be found in previously published research [5, 30].

### Data analysis

Path analysis was used to explore the relationships between the elements found to limit *L*. *chinensis* growth and the other elements considered in the study. In these analyses, the measured levels of the elements C, N, P, K, S, Mn, Fe, Zn, Cu and Na correspond to the independent variables *x*1, *x*2, *x*3, *x*4, *x*5, *x*6, *x*7, *x*8, *x*9, and *x*10, respectively, and the restricted elements identified within the aboveground *L*. *chinensis* samples correspond to the dependent variable y. The Amos 19.0 program was used to verify the path analysis coefficients and to draw path coefficient charts. Multi-element homeostasis relationships were quantified using the homeostasis model ($y=cx^{\frac{1}{H}}$) in Matlab [5] (R2016a, The Math Works Inc. Natick, MA). The results were visualized using Sigmaplot 12.5 (Systat Software, Inc. Germany).

## Results

### Identifying growth-limiting elements

To characterize the differences between the soda saline-alkali grasslands of the study area and other grasslands in China, the N and P contents and the N: P ratios of three representative grasslands were compared (Table 2). Independently of the degree of cover, the N: P ratio of *L*. *chinensis* in the study area was lower than in the reference areas. The limiting element was N in all sites at the study area and in the Songnen grassland, but that in Inner Mongolia and Tibet was P. The N content of *L*. *chinensis* was lower than the average value for terrestrial plants in China (20.20 mg/kg).

**Table 2:** N and P contents, N: P ratios and growth-limiting elements in the aboveground parts of plants collected from the study area and the reference areas.

Area	Area attribute	N (mg/g)	P (mg/g)	N:P	Limiting element	Data source
Research area	100%-70% cover	13.69	1.52	8.99	N	Average testing values of N and P
	70%-40% cover	17.72	1.64	10.82	N	
	40%-10% cover	17.16	1.48	11.60	N	
Reference area	Songnen grassland	24.20	2.00	13.00	N	Song et al. [32]
	Inner Mongolia grassland	26.80	1.80	16.40	P	He et al. [33]
	Tibetan grassland	28.60	1.90	15.70	P	He et al. [34]
	Average value for terrestrial plants in China	20.20	1.50	16.30	P	Han et al. [35]

(Note: Plant growth is N-limited when the N: P ratio is below 14:1. At ratios between 14:l and 16:l, N or P may be limiting, or there may be N+P co-limitation. If the N: P ratio is above 16:1, plant growth is P-limited [31].)

### Path analysis of relationships between N and other nutrient elements with different levels of cover

Stepwise regression was used to study the relationship between the measured contents of N and other nutrient elements in *L*. *chinensis* representing the three previously defined vegetation coverage groups (Table 3).

**Table 3:** Stepwise regression of the relationships between N and other elements in *L*. *chinensis*.

Cover	Variables related to dependent variables	Non-standardized coefficient		Standard coefficient	t	Sig.
		B	Standard error			
100%-70%	(constant)	-6589.445	2263.390		-2.911	0.011
	S	9.036	1.789	0.583	5.051	0.000
	Zn	585.571	134.717	0.501	4.347	0.001
70%-40%	(constant)	-2049.263	2481.395		-0.826	0.418
	Cu	1531.872	344.465	0.557	4.447	0.000
	K	0.401	0.113	0.444	3.548	0.002
40%-10%	(constant)	-830.218	3012.029		-0.276	0.786
	K	0.556	0.129	0.630	4.311	0.001
	S	5.351	2.157	0.363	2.480	0.025

**Fig 2:**
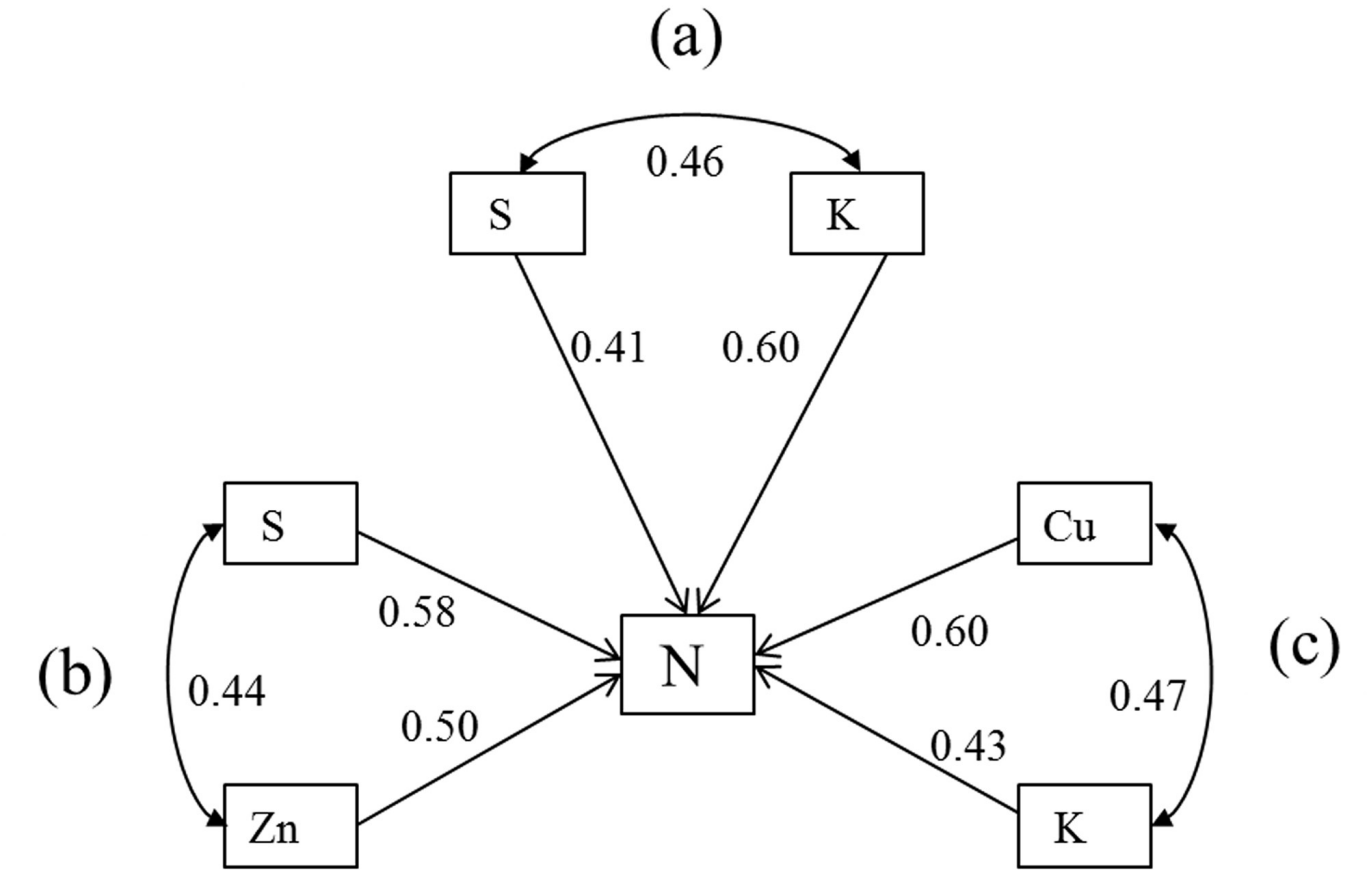
Path analysis results showing the relationships between the nutrient elements and the limiting element (N). (Note: (a) K, S, N path analysis for sites with 40%~10% coverage; (b) S, Zn, N path analysis for sites with 100%-70% coverage; (c) Cu, K, N path analysis for sites with 70%-40% coverage).

Elements whose calculated path coefficients indicated a correlation with the measured N content of the plant material were selected for further analysis, while the rest were excluded (Table 4 and Fig 2). S and Zn were found to be significantly correlated with N on saline-alkali grassland sites with 100%-70% cover; the path coefficient of S to N (0.58) was slightly higher than that of Zn to N (0.5) in this case. For sites with 70%-40% cover, Cu and K were correlated with N, but the path coefficient for Cu (0.60) was substantially greater than that for K (0.43). In sites with 40%-10% cover, K and S were correlated with N; the path coefficient of K to N (0.60) was much larger than that of S to N (0.41).

**Table 4:** Direct and indirect path coefficients between N and S, Zn, Cu, and K in *L*. *chinensis*.

Independent variable	Dependent variable	Correlation coefficient	Significance	Direct path coefficient	Indirect path coefficient					
					100%~70%		70%~40%		40%~10%	
					S	Zn	Cu	K	K	S
S	N	0.80	0.00	0.58	-	0.22	-	-	-	-
Zn		0.76	0.00	0.50	0.26	-	-	-	-	-
Cu	N	0.80	0.00	0.60	-	-	-	0.20	-	-
K		0.71	0.00	0.43	-	-	0.28	-	-	-
K	N	0.79	0.00	0.60	-	-	-	-	-	0.19
S		0.69	0.00	0.41	-	-	-	-	0.28	-

### Homeostatic characteristics of elements significantly related to N

**Fig 3:**
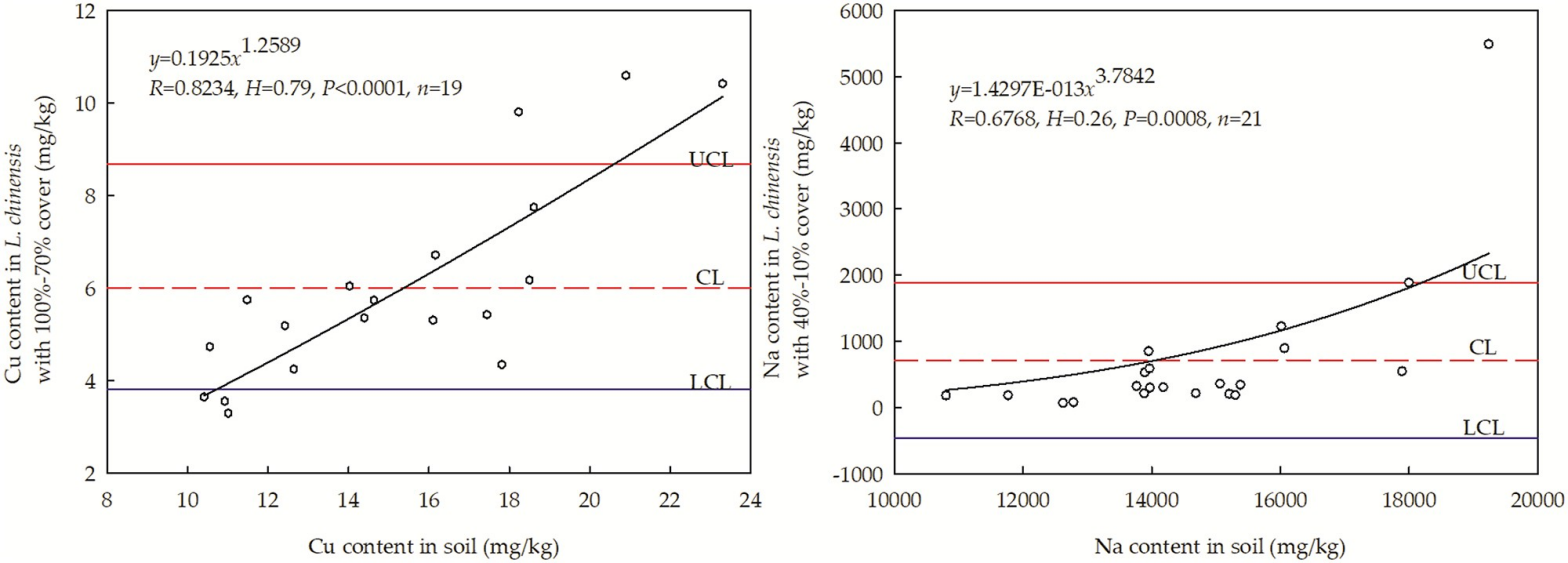
Homeostatic characteristics of two elements (Cu and Na) whose measured concentrations were significantly related to those of nitrogen in *L*. *chinensis*. (Note: The degree of homeostasis of an element in a given species is determined by its H-value; the species exhibits homeostasis of the element if H >4, weak homeostasis if 2 < H< 4, weak sensitivity if 4/3 < H < 2, and sensitivity if H < 4/3 [36]. The labels UCL, CL and LCL denote the upper control line, control line, and lower control line (based on a 95% confidence interval), respectively).

The above results reveal the elements that limit the N content of *L*. *chinensis* and the extent of N limitation as the degree of cover changes. Having identified these elements, their homeostasis in soda saline-alkali grasslands was investigated. For sites with 100%-70% cover, Cu (whose levels correlated significantly with those of N) exhibited the best fit to the homeostasis model, with an R of 0.8234. The homeostasis index value for Cu was 0.79, indicating that it should be classified as a “sensitive element.” In sites with 70%-40% cover, no element exhibiting strong correlation with N displayed any homeostatic characteristics. In highly degraded saline-alkali grassland sites with only 40%-10% cover, Na^+^, which can be detrimental to plant growth, exhibited homeostatic characteristics. However, its homeostasis index was 0.26, indicating that it too should be classified as a “sensitive element” in these sites (Fig 3).

## Discussion

The N contents of *L*. *chinensis* sampled in soda saline-alkali grasslands correlated significantly with their contents of S and Zn (Fig 2). S and N are both required for protein synthesis [37, 38], while Zn is a cofactor in many enzymes important for plant growth. In addition, many metal-chelating proteins have internal ligands featuring both N and S [39], suggesting that the relationship between these elements may be important for plant resistance to metal ion stress. Zn is an essential element involved in photosynthesis and promotes the synthesis of the growth hormones indoleacetic acid and tryptophan in combination with N [40]. The path coefficient of Zn and S is 0.44, indicating that the interaction between them is very strong, possibly because they are both required for the synthesis of zinc finger proteins. Numerous studies have highlighted the importance of these proteins for plant resistance to environmental stresses [41]. The correlation between Cu and N is more significant in saline-alkali grassland sites with 70%-40% cover. Cu is a metal with unique catalytic properties [42] that can form complexes with pigments and stabilize chlorophyll function [43] and can also help activate K-dependent stress resistance mechanisms that confer tolerance of saline-alkali stress and promote leaf growth in *L*. *chinensis*. The direct path coefficient of K and N was 0.43 in sites with 70%-40% cover and 0.6 in sites with 40%-10% cover. It is well known that N is an essential determinant of photosynthetic ability in plant leaves. Kunz et al. recently demonstrated that K^+^ transport protein activity can positively affect the structure and function of chloroplasts and promote photosynthesis [44]. In addition, many studies have shown that K can enhance plant stress resistance, enhance cellular responses to adverse environmental conditions, and enhance plant tolerance to challenging conditions such as saline-alkali stress and drought [45, 46]. The interaction between K and S may be due to the fact that the K^+^ channel is composed of amino acids, and S is an essential raw material for amino acid biosynthesis [47].

The homeostasis index of Cu in grassland sites with 100%-70% cover was 0.79, indicating that it should be classified as a sensitive element. These results showed that although there was considerable variation in the soil Cu content at the studied sites, the homeostatic mechanisms of *L*. *chinensis* ensured that the concentration of this element within the plant remained within a relatively narrow range of 5–7 mg/kg (Fig 3 (left)). The results presented here indicate that there are limits on the ability of *L*. *chinensis* to maintain Cu homeostasis, when the soil Cu content exceeded 19 mg/kg, the plants’ Cu homeostasis mechanisms appeared to be overwhelmed, resulting in pronounced variation in the measured Cu contents. In grassland sites with 70%-10% cover, there was no clear evidence of Cu homeostasis in *L*. *chinensis*. This may be due to the greater saline-alkali stress at these sites, which may reduce the metabolic functionality of the plants’ cells. It may be that when the content of Cu in *L*. *chinensis* becomes too high because its homeostatic mechanisms have been overwhelmed, the toxic effects of Cu start outweighing its beneficial effects, inhibiting the plant’s growth. While there was no clear evidence of K, S, or Zn homeostasis in *L*. *chinensis*, all three elements interacted significantly with N. Therefore, changes in the plants’ contents of these elements will affect their biochemical processes because of the correlation of these elements with N, weakening the growth-promoting effects of N.

On grassland sites with only 40–10% *L*. *chinensis* cover, the homeostasis index of Na (which was harmful to plant growth) was only 0.26, indicating that the plants had very little ability to control their Na contents under these conditions. The majority of the Na^+^ contents measured in *L*. *chinensis* samples in this work were within a 95% confidence interval (i.e. between the UCL and LCL) of the control line, demonstrating that *L*. *chinensis* has some ability to control its Na content. However, when the soil Na content exceeded 16000 mg/kg, the plants’ Na contents fluctuated greatly and became uncontrolled (Fig 3, (right)). This may have adversely affected the plants’ osmotic balance, causing loss of water and inactivation of plant cells. It is well established that excessive Na levels can have negative effects on cells’ biochemical processes [45], often causing irreversible damage to plants. Because *L*. *chinensis* cells exposed to high Na^+^ concentrations may lose the ability to grow. In accordance with this conclusion and the results presented here, Li Xia et al. showed that as the content of Na^+^ in soil increased, the species diversity and richness decreased [48]. To maintain Na homeostasis in *L*. *chinensis*, the content of Na in soils should be monitored and efforts should be made to ensure it does not exceed 16000 mg/kg.

To summarize, when grassland degradation was not serious, N promoted the growth of *L*. *chinensis* in combination with K, S, Cu, and Zn. However, *L*. *chinensis* was damaged by severe saline-alkali stress (i.e. high soil Na^+^ contents). However, more severe saline-alkali stress overwhelmed the regulatory mechanisms of *L*. *chinensis*, causing imbalances in the homeostasis of certain elements. Such homeostatic imbalances are particularly detrimental when they affect elements that interact significantly with N, and may restrict some of N’s physiological functions, leading to photosynthetic disorder, slow growth and low productivity. These defects then aggravate grassland degradation.

## Conclusions

The growth of *L*. *chinensis* at the studied sites was limited by N, and the elements that promoted N was K, S, Cu and Zn. In soils with Cu contents of 10~19 mg/kg, the content of Cu in *L*. *chinensis* remained stable in the range of 5~7 mg/kg. However, when the soil’s Cu content exceeded 19 mg/kg, the mechanisms that maintain Cu homeostasis in *L*. *chinensis* appeared to change or become overwhelmed, causing the plants’ Cu contents to fluctuate greatly. In saline-alkali grassland sites with very low levels of vegetation cover (40%-10%), there was evidence of weak homeostasis of Na^+^, which harms plant growth. When the soil’s Na content was in the range of 11000–16000 mg/kg, the homeostatic mechanisms of *L*. *chinensis* appeared to act as normal and to effectively regulate the plants’ Na contents. However, when the soil Na content exceeded the upper bound of this range, the plants’ Na contents increased rapidly, causing cells to lose water and become inactivated. Therefore, special attention should be paid to monitoring and regulating levels of Cu and Na in the soils of grasslands at risk of (further) degradation. In addition, when seeking to improve and fertilize degraded saline-alkali grasslands, particular attention should be paid to elements that had strong interactions with N and weak homeostasis. Elements of this type in sites with 100%-70% *L*. *chinensis* cover were S and Zn, while those in sites with 70%-40% cover and 40%-10% cover were K and Cu, and K and S, respectively.

## Supporting information

S1 File (DOC)
