# Supplementary material for: Cu and Na contents regulate N uptake of Leymus chinensis growing in soda saline-alkali soil
Source: PLoS One. 2020 Dec 1;15(12):e0243172. doi: 10.1371/journal.pone.0243172 (PMC7707461; doi:10.1371/journal.pone.0243172)
Supplement: S1 File — (DOC) [file pone.0243172.s001.doc]

100%-70% cover (*L. chinensis*)

| Number | mg/kg | mg/kg | mg/kg | mg/kg | mg/kg | mg/kg | mg/kg | mg/kg | mg/kg | mg/kg | mg/kg | mg/kg |
| --- | --- | --- | --- | --- | --- | --- | --- | --- | --- | --- | --- | --- |
|  | TN | Na | Mg | P | S | K | Ca | Mn | Fe | Ni | Cu | Zn |
| P13 | 21217.26 | 4608.78 | 982.06 | 1770.26 | 1544.45 | 17585.99 | 4195.56 | 60.43 | 508.68 | 1.40 | 10.60 | 33.68 |
| P14 | 16856.37 | 744.00 | 1611.41 | 1693.14 | 2807.27 | 19951.73 | 2399.19 | 27.88 | 286.17 | 2.88 | 9.81 | 18.15 |
| P15 | 14430.78 | 1606.45 | 1428.98 | 2085.78 | 1024.35 | 18498.01 | 3287.47 | 47.62 | 686.15 | 1.91 | 7.75 | 20.53 |
| P16 | 10717.00 | 1398.54 | 1112.55 | 1845.52 | 927.44 | 16995.72 | 2474.50 | 48.99 | 369.44 | 1.39 | 10.42 | 16.08 |
| P17 | 10615.93 | 1412.52 | 748.63 | 1425.44 | 902.83 | 18846.06 | 2900.65 | 40.39 | 437.56 | 1.07 | 6.05 | 15.06 |
| P18 | 10724.23 | 533.84 | 818.21 | 1473.80 | 1016.13 | 18713.39 | 2898.12 | 48.33 | 282.25 | 1.07 | 5.31 | 16.25 |
| P19 | 8543.57 | 1400.52 | 774.95 | 1825.03 | 881.05 | 16045.91 | 2699.65 | 33.85 | 438.84 | 0.75 | 6.18 | 14.35 |
| P23 | 9505.84 | 654.63 | 904.30 | 1318.80 | 918.98 | 17024.82 | 1558.74 | 33.25 | 246.63 | 0.61 | 5.75 | 16.32 |
| P40 | 7905.22 | 3544.51 | 883.62 | 899.39 | 737.41 | 10234.03 | 3242.21 | 28.27 | 255.68 | 0.41 | 3.65 | 11.55 |
| P41 | 11718.05 | 459.04 | 1700.48 | 1015.91 | 834.47 | 10560.65 | 3820.52 | 29.53 | 224.29 | 0.96 | 3.56 | 15.09 |
| P42 | 11646.44 | 215.76 | 1267.90 | 1095.84 | 827.94 | 14778.81 | 3570.36 | 33.93 | 241.97 | 0.29 | 3.30 | 14.88 |
| P49 | 10643.81 | 876.28 | 1224.89 | 1316.78 | 1144.87 | 22241.69 | 1716.78 | 10.02 | 218.19 | 0.34 | 5.19 | 12.78 |
| P50 | 13668.07 | 796.03 | 899.23 | 1666.31 | 1241.81 | 23564.90 | 1097.53 | 3.37 | 210.39 | 0.33 | 4.74 | 21.37 |
| P51 | 15261.87 | 690.35 | 1014.80 | 2117.64 | 1628.88 | 24447.57 | 1197.50 | 4.08 | 182.00 | 0.29 | 4.25 | 12.49 |
| P57 | 19724.03 | 219.70 | 983.21 | 1868.20 | 1366.65 | 25113.43 | 3099.21 | 27.27 | 218.07 | 1.45 | 5.36 | 20.81 |
| P58 | 23073.32 | 745.88 | 1417.12 | 1757.08 | 1520.92 | 25485.73 | 3548.04 | 23.58 | 315.08 | 2.63 | 6.72 | 23.90 |
| P65 | 13261.42 | 520.26 | 1405.87 | 1276.98 | 1234.72 | 18808.19 | 4248.19 | 32.96 | 278.19 | 0.69 | 5.74 | 16.36 |
| P66 | 15230.77 | 989.32 | 1226.02 | 1101.00 | 1205.12 | 18130.42 | 3382.49 | 31.67 | 231.45 | 0.87 | 5.43 | 18.00 |
| P67 | 15275.99 | 358.00 | 1461.64 | 1355.40 | 1211.34 | 18418.79 | 4408.62 | 29.50 | 239.27 | 0.73 | 4.35 | 17.10 |

100%-70% cover (soil)

| Number | mg/kg | mg/kg | mg/kg | mg/kg | mg/kg | mg/kg | mg/kg | mg/kg | mg/kg | mg/kg | mg/kg | mg/kg |
| --- | --- | --- | --- | --- | --- | --- | --- | --- | --- | --- | --- | --- |
|  | TN1 | Na1 | Mg1 | P1 | S1 | K1 | Ca1 | Mn1 | Fe1 | Ni1 | Cu1 | Zn1 |
| S13 | 880.99 | 18304.66 | 5763.68 | 303.53 | 150.70 | 26883.63 | 35170.00 | 358.33 | 16853.41 | 28.05 | 20.90 | 43.79 |
| S14 | 1082.44 | 15585.90 | 6455.32 | 353.80 | 229.40 | 25392.22 | 44440.04 | 362.23 | 19347.86 | 28.65 | 18.23 | 37.15 |
| S15 | 753.31 | 17121.22 | 6018.32 | 288.64 | 115.70 | 26398.02 | 36023.86 | 335.87 | 16989.94 | 24.38 | 18.61 | 40.24 |
| S16 | 1551.09 | 14448.04 | 7426.78 | 447.91 | 323.51 | 24713.54 | 48116.23 | 375.27 | 21928.01 | 30.28 | 23.30 | 53.92 |
| S17 | 467.92 | 20271.27 | 4035.06 | 178.67 | 52.49 | 29590.93 | 28120.15 | 271.15 | 13272.74 | 18.37 | 14.03 | 35.58 |
| S18 | 1893.71 | 16908.41 | 5311.47 | 400.73 | 200.06 | 26337.33 | 37808.86 | 343.89 | 16826.17 | 24.21 | 16.10 | 40.60 |
| S19 | 1811.23 | 16394.10 | 5591.85 | 437.64 | 253.28 | 25235.38 | 42628.44 | 368.52 | 17960.54 | 26.81 | 18.50 | 45.76 |
| S23 | 1230.11 | 19014.79 | 4517.85 | 270.03 | 94.89 | 27682.72 | 30937.42 | 291.83 | 13417.84 | 18.72 | 11.48 | 28.79 |
| S40 | 725.37 | 14846.76 | 2869.09 | 168.48 | 55.72 | 28725.64 | 19441.38 | 199.01 | 9723.24 | 14.30 | 10.41 | 24.16 |
| S41 | 736.59 | 14325.54 | 2423.15 | 167.06 | 37.41 | 28639.10 | 14978.95 | 171.17 | 8589.57 | 14.09 | 10.92 | 25.58 |
| S42 | 1250.92 | 13725.75 | 2962.99 | 230.38 | 88.73 | 28659.53 | 18229.95 | 182.35 | 9430.00 | 14.67 | 11.01 | 26.02 |
| S49 | 713.69 | 18521.44 | 5118.40 | 345.54 | 325.85 | 25397.64 | 53135.54 | 306.22 | 13285.22 | 19.17 | 12.42 | 28.94 |
| S50 | 633.32 | 18282.03 | 5397.89 | 237.75 | 280.03 | 25628.00 | 46858.39 | 354.12 | 14247.66 | 20.27 | 10.55 | 25.60 |
| S51 | 724.24 | 13234.67 | 5284.63 | 216.92 | 303.55 | 21580.98 | 59292.62 | 303.52 | 13793.36 | 20.86 | 12.64 | 28.17 |
| S57 | 1928.67 | 14390.65 | 4326.40 | 365.73 | 373.88 | 23474.87 | 52918.36 | 268.26 | 12507.78 | 21.22 | 14.39 | 30.08 |
| S58 | 1149.99 | 14188.50 | 4581.51 | 272.37 | 352.06 | 23263.54 | 63814.08 | 266.46 | 13158.06 | 25.00 | 16.16 | 27.20 |
| S65 | 1420.59 | 16153.92 | 5020.99 | 345.41 | 300.07 | 24709.65 | 39193.49 | 320.23 | 15098.27 | 21.45 | 14.63 | 26.17 |
| S66 | 1025.80 | 16950.53 | 5806.18 | 286.21 | 306.20 | 25228.09 | 43023.33 | 328.45 | 14670.40 | 21.69 | 17.44 | 32.91 |
| S67 | 1396.54 | 15663.77 | 5378.13 | 333.16 | 339.17 | 23864.45 | 47847.36 | 318.16 | 15232.62 | 24.28 | 17.81 | 35.65 |

70%-40% cover (*L. chinensis*)

| Number | mg/kg | mg/kg | mg/kg | mg/kg | mg/kg | mg/kg | mg/kg | mg/kg | mg/kg | mg/kg | mg/kg | mg/kg |
| --- | --- | --- | --- | --- | --- | --- | --- | --- | --- | --- | --- | --- |
|  | TN | Na | Mg | P | S | K | Ca | Mn | Fe | Ni | Cu | Zn |
| P7 | 26092.02 | 296.06 | 1053.36 | 2140.03 | 1620.99 | 32097.83 | 3335.76 | 36.91 | 1436.48 | 1.74 | 8.47 | 26.84 |
| P8 | 27735.47 | 226.18 | 1225.79 | 2095.79 | 1611.65 | 35204.33 | 4014.17 | 28.96 | 629.27 | 1.07 | 13.22 | 30.95 |
| P9 | 18464.62 | 424.04 | 998.54 | 1587.87 | 1302.44 | 32030.68 | 3669.22 | 27.21 | 402.81 | 0.58 | 5.23 | 17.50 |
| P10 | 21924.27 | 1723.77 | 1217.48 | 2292.50 | 1659.78 | 21512.92 | 2944.57 | 37.47 | 646.54 | 1.71 | 8.49 | 19.01 |
| P11 | 27065.74 | 3899.12 | 1198.16 | 1881.46 | 3640.06 | 20981.45 | 2560.48 | 59.82 | 375.59 | 2.38 | 11.35 | 23.14 |
| P12 | 27395.58 | 1461.50 | 1969.30 | 2188.22 | 3283.21 | 22128.26 | 2162.36 | 35.26 | 341.35 | 2.07 | 11.70 | 25.90 |
| P20 | 10711.37 | 574.74 | 826.58 | 1536.11 | 997.63 | 18957.84 | 2940.63 | 59.66 | 458.66 | 0.80 | 5.31 | 15.70 |
| P21 | 8836.36 | 2119.05 | 790.07 | 1379.06 | 915.13 | 16152.49 | 2066.09 | 27.64 | 302.79 | 0.77 | 5.12 | 16.25 |
| P22 | 11356.60 | 827.77 | 1083.76 | 1374.23 | 1232.58 | 16598.52 | 1970.16 | 29.72 | 249.90 | 0.62 | 5.36 | 11.62 |
| P27 | 13715.10 | 5209.13 | 2260.02 | 2430.45 | 1035.09 | 27639.61 | 7451.30 | 20.78 | 195.84 | 1.59 | 6.06 | 17.89 |
| P28 | 5937.40 | 1350.55 | 682.74 | 935.68 | 482.66 | 5713.71 | 3349.83 | 52.97 | 806.24 | 1.20 | 4.42 | 14.71 |
| P29 | 12469.05 | 256.43 | 1293.50 | 1073.75 | 1870.70 | 15978.98 | 3175.41 | 34.69 | 545.82 | 0.72 | 7.54 | 9.12 |
| P33 | 22257.78 | 207.19 | 1331.06 | 1880.62 | 2055.42 | 28773.19 | 5273.45 | 36.45 | 520.43 | 1.47 | 8.88 | 19.98 |
| P34 | 18013.79 | 203.81 | 1494.76 | 1646.73 | 1483.43 | 19976.36 | 3174.36 | 25.12 | 282.03 | 2.03 | 8.20 | 27.88 |
| P37 | 15316.44 | 340.01 | 1103.28 | 1800.86 | 1204.03 | 18320.19 | 4429.36 | 19.74 | 377.71 | 0.94 | 8.00 | 14.68 |
| P38 | 19415.00 | 279.78 | 1324.89 | 1757.54 | 1432.38 | 21810.68 | 5512.53 | 21.87 | 321.55 | 0.86 | 7.04 | 16.48 |
| P39 | 11579.68 | 738.67 | 1324.91 | 889.68 | 669.14 | 12562.19 | 2547.08 | 30.56 | 239.91 | 0.36 | 5.41 | 12.14 |
| P46 | 12297.73 | 269.03 | 1037.49 | 1021.72 | 1002.03 | 14364.61 | 5515.14 | 20.45 | 258.00 | 2.76 | 4.41 | 14.43 |
| P47 | 12920.71 | 281.41 | 648.97 | 1044.38 | 1126.47 | 21056.37 | 2682.01 | 10.71 | 203.23 | 0.38 | 4.47 | 10.87 |
| P48 | 12511.05 | 769.56 | 823.81 | 1528.01 | 934.58 | 21484.43 | 1141.14 | 4.22 | 397.53 | 0.33 | 4.53 | 11.31 |
| P54 | 17471.75 | 302.36 | 1097.78 | 1903.65 | 1530.26 | 25202.61 | 3135.88 | 31.78 | 635.90 | 6.11 | 7.96 | 16.99 |
| P55 | 22819.81 | 343.65 | 1103.65 | 1968.62 | 1751.35 | 27639.14 | 3430.77 | 25.08 | 310.23 | 1.50 | 7.25 | 16.57 |
| P56 | 29342.61 | 440.16 | 1312.78 | 1961.96 | 1814.77 | 31790.65 | 3917.62 | 44.71 | 354.59 | 0.95 | 7.26 | 20.03 |
| P62 | 21075.18 | 4333.19 | 1188.44 | 1471.44 | 1503.07 | 15753.35 | 2791.23 | 36.26 | 250.24 | 0.77 | 7.68 | 17.82 |
| P63 | 19196.14 | 2018.90 | 1702.09 | 1336.88 | 1714.16 | 17904.71 | 4419.34 | 53.37 | 432.96 | 1.30 | 6.72 | 24.15 |
| P64 | 14777.68 | 453.73 | 1586.33 | 1468.92 | 1295.85 | 19068.39 | 5209.60 | 32.48 | 328.44 | 0.92 | 4.97 | 17.45 |

70%-40% cover (soil)

| Number | mg/kg | mg/kg | mg/kg | mg/kg | mg/kg | mg/kg | mg/kg | mg/kg | mg/kg | mg/kg | mg/kg | mg/kg |
| --- | --- | --- | --- | --- | --- | --- | --- | --- | --- | --- | --- | --- |
|  | TN1 | Na1 | Mg1 | P1 | S1 | K1 | Ca1 | Mn1 | Fe1 | Ni1 | Cu1 | Zn1 |
| S7 | 987.89 | 14718.03 | 4408.05 | 298.57 | 78.73 | 24641.62 | 23969.30 | 331.90 | 16207.02 | 24.66 | 16.95 | 41.04 |
| S8 | 1576.64 | 14972.58 | 4628.04 | 382.86 | 150.00 | 24887.44 | 25276.44 | 368.94 | 17067.26 | 28.90 | 20.81 | 43.41 |
| S9 | 2590.94 | 14286.77 | 4625.73 | 491.95 | 185.45 | 23780.93 | 23949.01 | 348.46 | 16812.81 | 22.77 | 18.37 | 38.93 |
| S10 | 431.72 | 19450.90 | 5469.83 | 247.02 | 129.88 | 26743.01 | 29909.16 | 328.23 | 15674.98 | 20.75 | 15.03 | 30.75 |
| S11 | 359.70 | 18518.61 | 5291.65 | 284.19 | 149.30 | 26721.90 | 34294.16 | 340.74 | 16332.25 | 26.04 | 18.52 | 38.51 |
| S12 | 818.05 | 20640.22 | 5327.98 | 212.87 | 98.08 | 26871.30 | 32318.20 | 351.72 | 15486.54 | 25.08 | 17.63 | 31.91 |
| S20 | 1837.57 | 16744.16 | 5237.65 | 445.06 | 276.92 | 26159.03 | 39391.90 | 357.29 | 17291.24 | 26.31 | 17.31 | 51.49 |
| S21 | 803.92 | 19575.79 | 4966.74 | 275.08 | 84.52 | 26956.80 | 32064.54 | 306.53 | 14750.90 | 24.24 | 20.48 | 37.41 |
| S22 | 1170.84 | 19213.38 | 5332.50 | 273.15 | 124.11 | 27811.95 | 34097.01 | 330.61 | 15784.20 | 20.56 | 15.21 | 43.47 |
| S27 | 969.05 | 20057.38 | 6974.68 | 323.54 | 322.86 | 24386.45 | 48490.51 | 356.67 | 18091.54 | 31.23 | 19.30 | 44.64 |
| S28 | 1152.25 | 11975.06 | 9610.90 | 400.93 | 314.78 | 23297.94 | 61788.41 | 445.08 | 27797.17 | 40.38 | 27.25 | 54.89 |
| S29 | 1041.57 | 13001.72 | 7381.82 | 369.27 | 316.01 | 23601.06 | 60269.20 | 355.03 | 17869.99 | 31.61 | 19.22 | 39.00 |
| S33 | 609.38 | 17805.00 | 2130.55 | 169.45 | 16.91 | 35414.64 | 9091.56 | 154.02 | 8080.17 | 11.94 | 10.08 | 22.09 |
| S34 | 2436.13 | 17302.70 | 3162.24 | 234.51 | 85.80 | 28019.18 | 12368.61 | 214.46 | 10192.72 | 14.32 | 11.97 | 29.97 |
| S37 | 478.00 | 15503.92 | 1889.04 | 146.49 | 14.30 | 29607.50 | 7396.60 | 166.45 | 7833.30 | 7.41 | 7.90 | 20.52 |
| S38 | 582.60 | 13494.85 | 1545.37 | 117.06 | 7.64 | 28756.69 | 5656.40 | 141.76 | 6745.10 | 9.63 | 7.85 | 15.03 |
| S39 | 813.47 | 14672.59 | 2697.56 | 175.57 | 47.54 | 27719.00 | 15939.58 | 207.97 | 9659.56 | 11.51 | 8.73 | 27.18 |
| S46 | 1579.16 | 16749.02 | 4583.31 | 295.26 | 131.43 | 27897.84 | 20126.50 | 253.59 | 15879.70 | 21.02 | 23.88 | 41.59 |
| S47 | 1358.90 | 17199.40 | 2774.95 | 171.93 | 28.39 | 28262.37 | 11183.89 | 186.26 | 10881.89 | 11.83 | 9.80 | 28.01 |
| S48 | 450.69 | 19926.47 | 4587.58 | 217.40 | 177.95 | 28203.12 | 37444.94 | 352.79 | 13328.00 | 16.57 | 10.37 | 30.27 |
| S54 | 2390.61 | 15437.77 | 3631.71 | 316.24 | 264.04 | 24435.13 | 37481.26 | 257.12 | 11271.97 | 18.74 | 14.65 | 31.37 |
| S55 | 2470.60 | 14794.28 | 3683.50 | 360.08 | 278.30 | 24866.77 | 34777.69 | 260.11 | 11531.17 | 17.68 | 13.99 | 24.83 |
| S56 | 2039.40 | 17030.51 | 3832.69 | 350.07 | 288.19 | 27411.42 | 33821.51 | 291.09 | 12202.78 | 21.34 | 17.42 | 43.43 |
| S62 | 1965.63 | 14714.96 | 4542.97 | 317.79 | 290.60 | 23528.71 | 36435.82 | 297.08 | 13582.72 | 15.33 | 12.11 | 28.22 |
| S63 | 1724.19 | 15170.06 | 4285.71 | 298.22 | 261.59 | 24544.55 | 29144.27 | 293.86 | 13388.90 | 17.44 | 11.84 | 91.50 |
| S64 | 1346.01 | 16389.61 | 5351.02 | 301.93 | 343.72 | 25179.35 | 45151.40 | 310.93 | 14571.20 | 20.45 | 15.37 | 29.32 |

40%-10% cover (*L. chinensis*)

| Number | mg/kg | mg/kg | mg/kg | mg/kg | mg/kg | mg/kg | mg/kg | mg/kg | mg/kg | mg/kg | mg/kg | mg/kg |
| --- | --- | --- | --- | --- | --- | --- | --- | --- | --- | --- | --- | --- |
|  | TN | Na | Mg | P | S | K | Ca | Mn | Fe | Ni | Cu | Zn |
| P1 | 15933.83 | 75.09 | 1567.16 | 1667.65 | 1460.48 | 18458.86 | 7106.11 | 73.26 | 835.57 | 2.60 | 15.52 | 22.25 |
| P2 | 19656.78 | 66.60 | 1751.68 | 1846.93 | 1442.18 | 18567.02 | 5167.81 | 58.56 | 2301.99 | 3.53 | 11.59 | 33.05 |
| P3 | 27608.83 | 180.12 | 2098.72 | 3804.86 | 2001.18 | 29001.42 | 4616.22 | 33.47 | 541.92 | 1.58 | 7.96 | 17.21 |
| P4 | 17891.28 | 178.15 | 964.74 | 1744.41 | 1099.87 | 15511.60 | 3299.17 | 24.22 | 924.47 | 3.13 | 6.01 | 14.33 |
| P5 | 23055.79 | 202.03 | 911.28 | 1874.48 | 1414.93 | 28796.34 | 3301.04 | 26.99 | 510.69 | 1.10 | 10.60 | 18.10 |
| P6 | 27416.46 | 183.83 | 1172.16 | 2110.44 | 1759.44 | 34338.28 | 3597.12 | 50.24 | 754.14 | 1.08 | 8.09 | 40.64 |
| P24 | 8695.04 | 892.81 | 782.52 | 1433.12 | 887.76 | 15582.47 | 3602.68 | 43.81 | 441.61 | 2.02 | 6.85 | 16.49 |
| P25 | 10468.00 | 1885.86 | 840.88 | 1452.00 | 862.35 | 18928.21 | 1800.17 | 22.89 | 245.12 | 0.48 | 4.74 | 16.68 |
| P26 | 10493.11 | 5493.48 | 916.26 | 1458.73 | 939.76 | 12859.23 | 1956.45 | 43.67 | 381.72 | 1.79 | 5.73 | 20.29 |
| P30 | 17919.68 | 339.94 | 1444.27 | 1391.10 | 2082.89 | 17424.10 | 3540.40 | 41.73 | 337.01 | 1.92 | 8.07 | 10.16 |
| P31 | 14553.92 | 212.08 | 1104.61 | 1026.78 | 1368.38 | 13669.52 | 2607.55 | 22.87 | 353.98 | 0.81 | 5.67 | 9.85 |
| P32 | 12870.21 | 302.10 | 1271.60 | 1045.78 | 1828.38 | 15894.64 | 2675.87 | 43.50 | 212.07 | 0.78 | 6.98 | 7.68 |
| P35 | 20198.16 | 295.63 | 1242.37 | 1338.37 | 1610.88 | 16281.55 | 6055.65 | 54.60 | 302.27 | 1.99 | 7.90 | 12.13 |
| P36 | 13814.38 | 525.86 | 1186.73 | 1251.86 | 1158.62 | 13530.79 | 4374.50 | 19.25 | 394.80 | 1.11 | 7.35 | 17.16 |
| P43 | 16907.46 | 588.20 | 1488.61 | 1279.41 | 1153.46 | 15453.64 | 3076.63 | 28.36 | 225.48 | 0.73 | 6.33 | 19.90 |
| P44 | 15866.73 | 1225.62 | 1098.83 | 955.34 | 1038.13 | 15502.80 | 1479.78 | 19.01 | 176.87 | 1.07 | 4.78 | 7.85 |
| P45 | 14556.81 | 543.69 | 986.21 | 1375.98 | 1126.58 | 18028.01 | 2074.74 | 12.85 | 205.22 | 0.31 | 5.46 | 12.60 |
| P52 | 24653.49 | 209.81 | 1024.26 | 1815.89 | 1609.99 | 23836.31 | 2873.45 | 33.91 | 354.44 | 1.45 | 10.80 | 19.36 |
| P53 | 25813.81 | 355.70 | 1000.57 | 1724.50 | 1679.04 | 27201.10 | 2924.95 | 29.21 | 522.44 | 1.39 | 8.08 | 19.61 |
| P59 | 15209.29 | 848.07 | 887.31 | 1214.91 | 946.09 | 18361.17 | 2896.50 | 24.36 | 461.18 | 0.48 | 10.98 | 11.95 |
| P60 | 16704.09 | 321.68 | 1073.37 | 1752.59 | 1295.46 | 25672.98 | 3268.74 | 21.48 | 351.22 | 1.25 | 7.20 | 24.55 |
| P61 | 17734.88 | 5778.46 | 1299.17 | 1313.02 | 1519.89 | 13705.85 | 2699.21 | 37.50 | 229.08 | 0.40 | 6.86 | 15.27 |

40%-10% cover (soil)

| Number | mg/kg | mg/kg | mg/kg | mg/kg | mg/kg | mg/kg | mg/kg | mg/kg | mg/kg | mg/kg | mg/kg | mg/kg |
| --- | --- | --- | --- | --- | --- | --- | --- | --- | --- | --- | --- | --- |
|  | TN1 | Na1 | Mg1 | P1 | S1 | K1 | Ca1 | Mn1 | Fe1 | Ni1 | Cu1 | Zn1 |
| S1 | 1586.83 | 12783.01 | 3098.36 | 157.54 | 135.44 | 22943.31 | 6585.05 | 334.95 | 14844.89 | 22.11 | 16.06 | 34.65 |
| S2 | 1393.25 | 12621.44 | 2866.86 | 211.95 | 82.87 | 23620.50 | 6118.71 | 321.68 | 15204.63 | 16.14 | 10.36 | 24.94 |
| S3 | 1278.99 | 11767.13 | 3353.12 | 255.75 | 97.71 | 23153.55 | 6984.60 | 335.99 | 15991.02 | 15.49 | 11.41 | 35.47 |
| S4 | 1390.93 | 10802.89 | 2334.65 | 179.98 | 31.74 | 21782.05 | 5006.44 | 243.88 | 11547.44 | 19.27 | 12.44 | 31.27 |
| S5 | 1510.92 | 15204.70 | 4409.07 | 380.79 | 45.78 | 25562.89 | 22209.44 | 341.81 | 16290.56 | 21.89 | 19.38 | 43.03 |
| S6 | 1394.47 | 15297.20 | 4133.24 | 335.34 | 120.32 | 24739.85 | 21716.92 | 340.35 | 15746.71 | 22.52 | 17.64 | 41.81 |
| S24 | 1444.00 | 16062.56 | 5974.89 | 414.02 | 201.63 | 26579.24 | 39242.61 | 355.35 | 18197.73 | 25.79 | 17.47 | 42.19 |
| S25 | 1160.57 | 17997.69 | 5387.80 | 315.34 | 241.55 | 27217.63 | 42959.75 | 317.38 | 15372.53 | 30.93 | 22.85 | 44.23 |
| S26 | 743.93 | 19234.17 | 5942.35 | 257.20 | 237.27 | 26005.22 | 46125.55 | 335.25 | 15901.59 | 22.18 | 15.39 | 31.24 |
| S30 | 809.23 | 15378.10 | 6503.29 | 298.12 | 342.41 | 24662.69 | 56200.38 | 328.94 | 16882.92 | 30.02 | 20.35 | 52.62 |
| S31 | 551.96 | 14684.62 | 7331.99 | 239.05 | 466.68 | 22510.72 | 71563.35 | 286.22 | 15253.85 | 29.05 | 20.28 | 48.87 |
| S32 | 1305.97 | 14179.03 | 6227.04 | 325.11 | 384.41 | 24142.82 | 57910.02 | 337.51 | 15822.38 | 25.84 | 18.43 | 42.56 |
| S35 | 674.07 | 13971.90 | 1736.82 | 129.57 | 11.90 | 29412.34 | 5025.94 | 162.69 | 7772.81 | 10.46 | 7.89 | 15.02 |
| S36 | 755.73 | 13892.36 | 1872.70 | 140.27 | 12.84 | 27903.87 | 6628.97 | 159.17 | 7946.58 | 10.44 | 8.60 | 20.00 |
| S43 | 915.07 | 13965.18 | 4096.88 | 249.43 | 168.72 | 27532.25 | 24759.72 | 211.48 | 10597.47 | 18.84 | 12.74 | 27.80 |
| S44 | 763.88 | 16013.29 | 6734.62 | 298.17 | 312.45 | 25130.11 | 48243.44 | 297.38 | 15001.27 | 17.57 | 12.10 | 27.90 |
| S45 | 2148.80 | 17889.46 | 3564.22 | 324.45 | 190.22 | 27465.22 | 18097.78 | 267.70 | 12097.04 | 13.49 | 10.16 | 26.88 |
| S52 | 2598.13 | 13882.54 | 4133.95 | 423.80 | 353.87 | 23524.91 | 48302.10 | 264.76 | 12566.22 | 21.05 | 14.20 | 31.04 |
| S53 | 2299.67 | 15061.47 | 3833.68 | 394.30 | 349.29 | 24179.07 | 33939.69 | 270.11 | 11435.25 | 17.87 | 13.69 | 31.27 |
| S59 | 1661.82 | 13951.30 | 4356.37 | 281.58 | 317.10 | 22857.36 | 52289.22 | 263.11 | 12658.90 | 22.30 | 15.76 | 30.36 |
| S60 | 2056.70 | 13764.71 | 4469.90 | 314.73 | 389.04 | 22276.38 | 58721.42 | 251.84 | 11855.20 | 21.44 | 17.28 | 29.21 |
| S61 | 1738.30 | 15242.60 | 4344.46 | 322.37 | 247.59 | 23966.49 | 29357.45 | 310.50 | 14421.18 | 19.44 | 15.99 | 29.04 |
